# Notes of a Case of Ovarian Dropsy, with the Appearances Presented on Post Mortem Examination

**Published:** 1845-08

**Authors:** Edward R. Squibb


*Notes of a Case of Ovarian Dropsy, with the appearances presented on Post Mortem Examination.* By Edward R. Squibb, M. D.

The following case is interesting, not simply as one of ovarian dropsy, a disease which is now so well known, but from the situation and condition of the parts shown by an examination, and the bearing of this condition upon the operations proposed for the cure of the disease, by the removal of the tumour.

For this, as well as for many other opportunities for study, afforded by numerous cases, the writer is indebted to his friend and preceptor, Professor T. D. Mütter, under whose notice it first came, about a month previous to its termination.

The diagnosis, prognosis, and only admissible treatment, (palliative) indicated at the time by Professor M., subsequent observation has shown to have been correct.

On the 12th of June, 1845, the patient was seen by Dr. M. and the writer.—She was a woman of small stature, 31 years old, with dark hair and eyes, pale, and very much emaciated Previous to this illness she had enjoyed an ordinary degree of health; six years had elapsed since she was married,—three of which were passed in widowhood,—and had buried an only child, about four years old. To the bodily fatigue and mental anxiety to which she was subjected on the occasion of the long illness of this child, she always attributed the commencement of her diseased condition. Her spirits and disposition were, however, always good; the failing powers of life bringing with them only their necessary and absolute changes, without the augmentation which so frequently arises from mental complications. The symptoms at this date were as follows:

A dull intermittent pain over the left lower portion of the abdomen, frequently aggravated or brought on by change of position, and accompanied by a dragging sensation when laying upon the right side. A marked hectic fever, with the early and late paroxysms of each day well pronounced. These paroxysms were attended by an excessive gastric irritation, with vomiting, which was often prolonged through the intervals between them, allowing nothing to remain upon the stomach, even forcing up the mucous and saliva as they accumulated there. This distressed the patient very much, and aggravated her condition by the consequent debility it caused. With this marked hectic, there was no diarrhœa—the irritation or inflammation having attacked the upper portion of the alimentary canal, in opposition to the usual course. The bowels were somewhat torpid, and her rest at night much disturbed by the nausea and vomiting. A constant colourless discharge from the vagina, with some hemorrhoidal tumours, also added much to the discomfort of the patient. The appetite was poor, even when the stomach was in a condition to receive food,—but the thirst was constant, although not admitting of a quantity of fluid at a time. The pulse was variable,—generally quick, frequent, and thready. The respiration was free, and the functions of the brain not notably deranged.

Upon as thorough a physical examination as the patient's condition would admit of, a large bi-lobed tumour was detected occupying the whole of the left iliac and hypogastric, with a part of the right iliac, umbilical and left lumbar regions. This tumour was somewhat flattened in front, extending principally in a transverse direction, and presenting a somewhat indistinct fluctuation. Rude handling gave the patient pain, and any examination left a soreness which continued for many days. There was slight tenderness all over the abdomen, increased at the epigastrium, where a burning heat was felt during the hectic paroxysms. This tumour first attracted the patient's attention about nine months previous to her death, being then about the size of an ordinary hen's egg. Previous to this there had been no pain of a degree or character to attract notice, and at this time she only experienced occasional achings in that region. Some weeks after, the tumour having increased, she applied for surgical advice, and was told by the gentleman whom, at that time, she consulted, that her disease was of a cancerous nature and only admitted of palliation. Subsequent to this examination, and probably brought on by it, although it was conducted with the greatest care and gentleness, she suffered an attack of what, from the symptoms, appears to have been peritonitis. From this she shortly recovered, and was able to walk about until the accession of hectic, which occurred about two months prior to her death.

In view of these symptoms, at the time when Professor Mütter was consulted, the diagnosis was in favour of an encysted dropsy of the left ovary, and the prognosis unfavourable, the case admitting only of palliation. At this time the case was entrusted to the writer for treatment. This consisted, for the first thirteen days, of cold infusion of wild cherry bark, ad libitum, small pieces of ice swallowed frequently, frictions with croton oil at the epigastrium, a milk and farinacious diet, always taken ice cold, and at regulated intervals, perfect rest in the horizontal position, and free ventilation,—occasional cool sponging being also enjoined. The bowels were opened every alternate day by enemata of arrow root and castor oil, and a vaginal injection of cold solution of alum was used twice a day.

By these means the paroxysms of hectic seemed to be abated in degree and frequency, and the extreme irritability of the stomach overcome to a considerable extent. This latter, however, only took place after many days' perseverance, and much distrust of the efficacy of the means adopted. The appetite gradually improved, the leucorrhœal discharge diminished, and the patient, at the expiration of nearly two weeks, was much more comfortable.

Animal broths were now permitted, and a teaspoonful of good wine, with sugar, was added to each glass of milk, continuing the iced infusion of wild cherry bark, and keeping up slight counter-irritation at the epigastrium by the use of the croton oil. The enemata were discontinued, and replaced by an occasional simple rhubarb pill, whose tonic effect added to the other measures, appeared to be beneficial, whilst the bowels were kept in a soluble and sufficiently free condition. A solution of one drop each of creosote and acetic acid, in one ounce of water, was given in teaspoonful doses, when the now somewhat rare nausea came on; and, as her rest was at times disturbed, a teaspoonful of the solution of sulphate of morphia was given. Thus for eight days more the patient's condition continued to improve slightly, and was as comfortable as circumstances would admit, the tumour, however, still increasing, but giving her little positive pain.

At the expiration of twenty-one days after the treatment had commenced, the patient received a severe fright from an outrage committed in the house where she was. Following this, and doubtless as a consequence of it, the symptoms were renewed in an aggravated form, and before any attempts to subdue them could be successful, circumstances obliged her to be removed, even in her precarious condition, to the distant house of a relative, in order to avoid being turned into the street. She was then attacked, in addition, by a colliquative diarrhœa, over which cold mucilaginous and opiated injections, cool fomentations, acetate of lead and opium, chalk mixture and extract of rhatany, preceded by laxative enemata, and tried in succession, seemed to have little control. As her powers seemed fast failing, soft toast and cream, wine whey, wine and new milk, etc., were ordered, after which the diarrhœa was somewhat abated for the last two days; but the debility and sinking continued gradually to wear away the remnant of life, as each receding pulse wave showed more plainly its approaching boundary, until the 11th of July, when her sufferings terminated almost insensibly in death.

A crucial incision through the abdominal parietes, twenty-five hours after death, exhibited the contents in the following condition. Extensive bands of organized lymph connected some of the viscera to the sides of the lower part of the cavity, indicating a preceding inflammation. On attempting to raise the great omentum it was found slightly adherent to the viscera, etc., below, on the right side; but upon the left side it could not be detached from the tumour or the parietes of the abdomen. The cavity of the peritoneum contained two or three ounces of limpid serum, quite clear and colour.ess. The bladder was contracted and forced down behind the symphysis pubis, and contained no urine. The tumour consisted of two enormous cysts lying beside each other, and firmly adherent. The largest of these was to the left side, filled the entire iliac, and projected across into the hypogastric region. It was inseparably attached to the walls of the abdomen, and the fascia covering the iliacus internus and psoas muscles, so that in endeavouring to dissect it off the muscles and fascia were cut, and the cyst itself ruptured. The omentum on this side had disappeared, either by absorption from the pressure and inflammation, or from being fused into the tissues of the walls of the abdomen. The sigmoid flexure of the colon was curiously twisted out of shape and place, and with the left transverse and descending portions, and the rectum, was firmly attached to, and compressed by the tumour, its walls appearing at some places to be composed of the mesocolon and mesentery. The head of the colon lay behind the right tumour, being adherent to and compressed by it. The entire large intestine was very much contracted, empty, and in a state of brown congestion. The right cyst, although appearing at first sight much smaller than the first, was found to extend behind the left one, and lower down, so as to fill the space between he rectum and uterus, surrounding the posterior half of the neck of the latter, and adherent also to the anterior part of the rectum. It, however, appeared to be of somewhat less capacity than the first, and did not rise so high in the cavity of the abdomen.

In the nich between these two cysts, or in front of their union, lay the uterus, to the right of the median line, and principally upon the right tumour. It was very much flattened antero-posteriorly, and the sides flattened out into thin edges by the compression. It had entirely lost its shape and position, so that it was not at first recognized. It was firmly attached by the posterior surface to both tumours, and could not be dissected off. The front surface was, however, free from adhesions, as were the right ovary and fallopian tube. The cavity was of the usual size, and not much changed in form, although the anterior and posterior walls were thinned out. The neck was hypertrophied and indurated, being larger in circumference than the body.

Only a portion of the left ovary could be found, and this in a softened and diseased condition, upon the upper and outer side of the left cyst, three and a half inches from the uterus. No remains of the ligament of the ovary or fallopian tube could be detected.

The small intestines had the appearance of congestion, were distended with flatus, and forced upward from their natural position.

The stomach was contracted and very much softened, and the liver was of a paler colour than it is ordinarily, and also very much softened.

The cysts were diaphanous, having much the appearance of bladders distended with fluid. They contained a transparent fluid of a colour rather darker than ordinary urine, in which white flocculi were floating. The whole quantity of fluid may be estimated at from thirty to forty ounces, probably nearer to the latter quantity.

The extreme heat of the weather, and the condition of the body, (it having been kept without ice,) prevented a farther and more minute examination, particularly as the principal object had been attained, namely, a knowledge of the situation and relations of the tumour.

